# Duration of exclusive breastfeeding in a Brazilian population: new determinants in a cohort study

**DOI:** 10.1186/1471-2393-14-175

**Published:** 2014-05-26

**Authors:** Tatiana O Vieira, Graciete O Vieira, Nelson F de Oliveira, Carlos M C Mendes, Elsa Regina J Giugliani, Luciana R Silva

**Affiliations:** 1 State University of Feira de Santana, Feira de Santana, Bahia, Brazil; 2 Federal University of Bahia, Rua Barão do Rio Branco, CEP 44001-205, 1499 Feira de Santana, Bahia, Brazil; 3 Federal University of Rio Grande do Sul, Rio Grande do Sul, Brazil

**Keywords:** Breast feeding, Child nutrition sciences, Health status indicators, Cohort studies, Survival analysis

## Abstract

**Background:**

Determinants of the duration of exclusive breastfeeding (EBF) differ in effect and magnitude across populations. The present study aimed to identify factors associated with discontinuation of EBF in a municipality in northeastern Brazil, including variables that have received little or no attention in previous literature.

**Methods:**

This cohort study involved 1,344 mother-child pairs selected from maternity hospitals in Feira de Santana, Bahia, Brazil. Subjects were followed up for 6 months through monthly home visits, and discontinuation of EBF was recorded. Possible determinants were tested using Cox’s four-level hierarchical survival model, taking into consideration the temporal proximity of the predisposing factors to interruption of EBF. Median duration of EBF was estimated using Kaplan-Meier’s survival curve.

**Results:**

Median duration of EBF was 89 days. Out of the 19 variables tested, 9 showed an association with EBF cessation; of these, two had never been evaluated in Brazilian studies, namely, mother partner’s appreciation for breastfeeding (hazard ratio [HR] 0.62; 95% confidence interval [95% CI] 0.48-0.79) and limiting the number of nighttime feeds at the breast (HR 1.58; 95% CI 1.11-2.23). Another two variables that had been previously evaluated, but had never been described as determinants of discontinuation of EBF showed association: presence of cracked nipples (HR 2.54; 95% CI 2.06-3.13) and prenatal care provided by public services (HR 1.34; 95% CI 1.17-1.55). Other variables showing associations with the outcome were: guidance on breastfeeding received at the hospital (HR 0.80; 95% CI 0.68-0.92), birth in a Baby-Friendly Hospital (HR 0.85; 95% CI 0.73-0.99), less than or equal to 8 years of maternal schooling (HR 1.34, 95% CI 1.17-1.53), mother working outside the home (HR 1.73; 95% CI 1.53-1.95), and use of a pacifier (HR 1.40; 95% CI 1.14-1.71).

**Conclusions:**

The study confirmed that the factors associated with EBF duration are multiple, variable, and dependent on the population being evaluated. Characteristics that had never been previously evaluated or described, at least in Brazilian studies, behaved as determinants of EBF in the present study, and thus allow to expand the existing list of factors determining this practice.

## Background

It is well known that breastfeeding, even in developed countries, protects against gastrointestinal and respiratory infection, sudden infant death syndrome, diabetes, allergy, and, in preterm infants, necrotizing enterocolitis [1]. It is also known that the protection conferred by breastfeeding is maximized with greater duration and exclusivity of breastfeeding.

Despite the scientific evidence recommending exclusive breastfeeding (EBF) for the first 6 months of life [2], this practice has low prevalence worldwide [3-7], including in Brazil [8,9]. A 2012 report on the global breastfeeding situation by the United Nations Children's Fund, with data from more than 120 countries, revealed that the prevalence of EBF in children under 6 months was 37% [10]. In Brazil, 95% of the mothers initiate breastfeeding [9], but the duration of EBF is still low. The most recent survey conducted in Brazil with a nationwide representative sample revealed that the median duration of EBF was 1.4 month [9]. There is great variability in the duration of EBF in the 27 Brazilian capitals: results range from 0.7 day in a capital of the central-western region to 88.8 days in one of the northern region [8]. This variation probably reflects the multifactorial nature of breastfeeding, regulated by biological, psychological, socioeconomic, ethical, and cultural factors. For this reason, there is a need to improve our knowledge of the factors with the greatest influence on the prevalence of breastfeeding in different localities.

The factors associated with early EBF cessation have been the subject of several studies conducted around the world [3-7,11-17]. Pacifier use, low maternal education, fathers’ preference for artificial feeding, maternal smoking during pregnancy, and problems with breastfeeding in the first month of life are some of the determinants reported in the literature [18,19]. A recent systematic review [20] of studies conducted in Brazil identified 47 determinants of EBF in 21 studies. The review found that some of the determinants had been investigated in over a third of the studies, namely, pacifier use, newborn birth weight and sex, maternal age, educational level, parity and work situation, prenatal care, birth in a Baby-Friendly Hospital, and type of delivery. Conversely, other variables had been little studied, e.g., mother’s intention regarding the duration of breastfeeding, public or private prenatal care, and guidance on breastfeeding received at the maternity hospital. Moreover, some determinants had never been described in Brazilian studies, e.g., mother limiting the number of nighttime feeds at the breast and mother partner’s appreciation for breastfeeding [20].

Among the factors that have been studied, the great variety of effects and magnitudes stands out, particularly for individual factors, such as newborn sex, mother’s age, educational level, work situation, and type of delivery, among others. In addition, the characteristics of the mother’s microenvironment, including those of the public healthcare service, may exacerbate or ameliorate the effects of the different individual factors. As a result, a given factor may be associated with the prevalence or duration of EBF in one locality, but not in another.

Thus, considering that the determinants of EBF differ among populations and that it is important to improve our knowledge of these determinants in order to plan actions aimed at increasing the duration of EBF, the objective of the present study was to identify factors associated with early EBF cessation in a city located in northeastern Brazil, including variables that have received little or no attention in previous literature.

## Methods

### Study design

This cohort study assessed live births in Feira de Santana, Bahia, a large-sized city in northeastern Brazil, located 108 km distant from the state capital (Salvador). The population of Feira de Santana in 2012 was around 568,000 inhabitants [21]. The city is an important economic center for commerce, industry, and livestock rearing.

### Sample size

Sample size calculation estimated that 1,265 children would be required for the study. This calculation took into consideration the number of live births in the municipality in 2003 (10,177), the proportion of children under EBF at 6 months of age reported in a previous study (38.5%) [22] a significance level of 5%, a test power of 90%, and a difference in the outcome of at least 5%, using finite population correction.

### Data collection

Mother-child pairs were recruited from all the 10 maternity hospitals in the city. Inclusion criteria were residing in the city and not having any contraindications to breastfeeding. Subjects entered the cohort over a 12-month period, between April 2004 and March 2005. Mothers from each hospital were recruited for the study over a period of 2 months; two hospitals were selected by draw every two months, except for two hospitals which were selected separately due to the large number of deliveries performed.

After signing a free, informed consent form, mothers were interviewed by trained healthcare professionals at the maternity hospital and then in their homes during monthly visits over the infant’s first 6 months of life. Of a total of 1,360 mother-child pairs considered eligible for the study, 1,344 were included in the cohort; 10 women refused to participate, 4 could not inform their address, and 2 lived in very dangerous areas.

### Variables

The outcome was defined as discontinuation of EBF within the first 6 months of the child’s life and was investigated every month during the home visits. On these occasions, mothers were asked about the foods that they had offered to their children over the 24-hour period preceding the interview: mother’s milk, another type of milk, water, tea, juice, fruits, and any other foods. The World Health Organization’s (WHO) definition [2] of EBF was used, i.e., children receiving only breast milk, with no intake of any other liquids or solids, except for medications.

**Figure 1:**
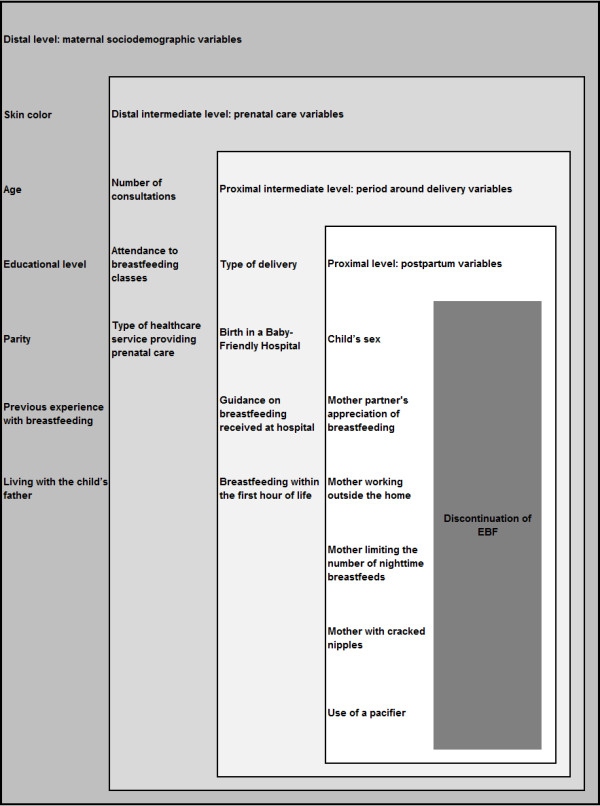
Theoretical hierarchical model for discontinuation of exclusive breastfeeding (EBF).

The independent variables were grouped into four levels, in accordance with a theoretical hierarchical model for discontinuation of EBF. In this model, predisposing factors are organized according to their proximity to the outcome [20] (Figure 1), as follows: individual sociodemographic factors (distal model); prenatal factors (distal intermediate model); factors relating to the period around delivery (proximal intermediate model); and postnatal factors (proximal model).

The following maternal sociodemographic characteristics were recorded: skin color (self-reported, white or non-white); age at delivery (<20 or ≥ 20 years); educational level (≤8 or > 8 years of schooling); parity (primiparous or multiparous), previous experience with breastfeeding (yes or no); and living with the child’s father (yes or no).

Characteristics of the gestational period were also investigated, as follows: number of prenatal consultations (<6 or ≥ 6 consultations – inadequate or adequate, respectively, according to the Brazilian Ministry of Health) [23]; attendance to prenatal breastfeeding classes (yes or no); and type of healthcare service providing prenatal care (private or public). Variables relating to the period around delivery were: type of delivery (vaginal or cesarean); birth in a Baby-Friendly Hospital (yes or no); guidance on breastfeeding received at the maternity hospital (yes or no); and breastfeeding within the first hour of life (yes or no).

During the postnatal period, the following variables were evaluated: child’s sex (male or female); mother partner’s appreciation for breastfeeding (yes or no), determined when the child was 30 days old by asking the mother whether the partner thought that breastfeeding was important. Some other data that might change over the course of time were obtained every month, e.g.: mother working outside the home (yes or no); limiting nighttime breastfeeding (yes or no); presence of cracked nipples (scratch or laceration on nipples as reported by the mother) (yes or no); and use of a pacifier (yes or no).

### Statistical analysis

The data collected were double-entered into the Statistical Package for the Social Sciences (SPSS) for Windows software, version 16.0 (Chicago, IL, USA) and validated. The SPSS 16.0 and R 15.1 statistical software were used for statistical analysis. Median duration of EBF was estimated using Kaplan-Meier survival analysis. To test associations between the outcome and the independent variables, the Cox extended multivariate model with fixed and time-varying covariates was used; the entry of predictive factors into the model was performed according to the hierarchical levels established. All variables at each level were adjusted in relation to each other, and those presenting statistically significant associations with the outcome at the 10% level were selected to remain in the next model. As a result, variables of the distal intermediate group were adjusted according to the eligible variables from the distal group, and so on successively, until the last group of variables (proximal group) was included, thereby reaching definition of the factors associated with discontinuation of EBF. In the final model, statistical significance was set at 5%.

It is important to mention that the model selection strategy used in this study focused on literature data and on biological plausibility. Initially, variables considered potentially relevant to describe EBF duration were selected for inclusion in the model; subsequently, backward automatic routines were used to select the variables that should remain in the final model. Before modeling completion, the possibility of including tow-factor interaction terms among the covariates present in the model was assessed, but no change in effect was observed. Model parameters were estimated using the maximum likelihood method, and related hypotheses were tested using the likelihood ratio test. The model was diagnosed and showed good adjustment. Proportional hazards were assessed using Schoenfeld’s residual tests, then the potential presence of atypical individuals (outliers) was investigated using Deviance residuals, and finally the influence exerted by each individual on several aspects of the adjusted model was assessed using Jackknife residuals. The analysis considered the possibility that predictors might vary over time, expressing different values at each interview.

### Ethical aspects

This investigation was approved by the Ethics and Research Committee of the State University of Feira de Santana, Bahia, Brazil (protocol no. 012/2003). All women included in the study provided informed consent.

## Results

### Study population and median duration of EBF

During the follow-up period, 83 pairs were lost while still being exclusively breastfed (6.2%). These pairs were censored at the last valid interview, as follows: 35 during the first month, and 27, 6, 10, and 5 in the second, third, fourth, and fifth months, respectively.

**Figure 2:**
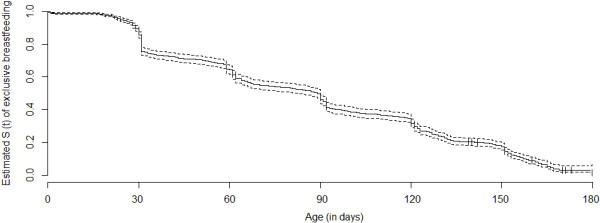
Kaplan-Meier’s survival curve for exclusive breastfeeding with 95% confidence intervals.

Infant characteristics are shown in Table 1. All children in the sample were breastfed on the first day of life. Median duration of EBF was 89 days (95% CI 80–90 days). Figure 2 shows the survival curve for the probability of EBF in the children’s first 6 months of life. On the first day of life and at the end of the first month, 97.3 and 89.6% of the children were being exclusively breastfed, respectively; conversely, only 11.3% (n = 148) were still being exclusively breastfed at the end of the study period.

**Table 1:** Characteristics of the cohort of 1,344 live births from Feira de Santana, Bahia

**Sample characteristics**	**Total N (%)**
**Maternal skin color**	
White	245 (18.2)
Non-white	1099 (81.8)
**Maternal age at delivery**	
< 20 years	262 (19.5)
≥ 20 years	1082 (80.5)
**Maternal educational level**	
≤ 8 years of schooling	508 (37.8)
> 8 years of schooling	836 (62.2)
**Parity**	
Primiparous	671 (49.9)
Multiparous	673 (50.1)
**Previous experience with breastfeeding**	
Yes	651 (48.4)
No	693 (51.6)
**Mother living with the child’s father**	
Yes	1145 (85.2)
No	199 (14.8)
**Number of prenatal consultations**	
< 6	352 (26.2)
≥ 6	992 (73.8)
**Attendance to prenatal breastfeeding class**	
Yes	347 (25.8)
No	997 (74.2)
**Prenatal care provided by public service**	
Yes	888 (66.1)
No	456 (33.9)
**Vaginal delivery**	
Yes	759 (56.5)
No	585 (43.5)
**Birth in a Baby-Friendly Hospital**	
Yes	343 (25.5)
No	1001 (74.5)
**Guidance on breastfeeding received at hospital**	
Yes	1094 (81.4)
No	250 (18.6)
**Breastfeeding within first hour of life**	
Yes	645 (48.0)
No	699 (52.0)
**Baby’s sex**	
Male	719 (53.3)
Female	625 (46.5)
**Mother partner’s appreciation for breastfeeding**	
Yes	1274 (94.7)
No	70 (5.3)
**Mother working outside home**	
Yes	138 (10.3)
No	1206 (89.7)
**Mother limiting number of nighttime breastfeeds**	
Yes	35 (2.7)
No	1309 (97.3)
**Presence of cracked nipples**	
Yes	159 (11.8)
No	1185 (88.2)
**Use of a pacifier**	
Yes	602 (44.8)
No	742 (55.2)

### Influence of sociodemographic, prenatal, delivery, and postpartum factors

Table 2 shows the crude and adjusted effects (hazard ratio) of each predictor on EBF cessation during the infant’s first 6 months of life. According to the theoretical hierarchical model proposed, the variable maternal education was selected from model 1 to continue in the analysis. From the second model, the variables number of prenatal consultations and type of healthcare service providing prenatal care were selected. Birth in a Baby-Friendly Hospital and guidance on breastfeeding received at the maternity hospital were the variables selected from model 3. Mother partner’s appreciation for breastfeeding, mother working outside the home, mother’s limiting nighttime feeds at the breast, presence of cracked nipples, and use of a pacifier were the variables selected from model 4. All these variables were included in the final model. Except for number of prenatal consultations, all the variables maintained their association with the outcome even after adjustment for the other variables.

**Table 2:** Cox analysis for the risk of exclusive breastfeeding cessation in cohort from Feira de Santana

**Variables**	**Distal model**	**Distal intermediate model**	**Proximal intermediate model**	**Proximal model**
	HR (95% CI)*	HR (95% CI)*	HR (95% CI)*	HR (95% CI)*
White mother	1.00 (0.85-1.17)	--	--	--
Maternal age < 20 years at delivery	0.96 (0.81-1.13)	--	--	--
Maternal educational level ≤ 8 years of schooling	1.51 (1.32-1.72)	1.31 (1.15-1.50)	1.32 (1.15-1.50)	1.34 (1.17-1.53)
Primiparous	0.95 (0.60-1.50)	--	--	--
Having previous experience with breastfeeding	0.76 (0.49-1.19)	--	--	--
Mother living with the child’s father	0.90 (0.76-1.06)	--	--	--
< 6 prenatal consultations attended	--	1.14 (0.98-1.31)	1.15 (1.00-1.33)	1.15 (1.00-1.33)
Prenatal breastfeeding class attended	--	0.96 (0.84-1.10)	--	--
Prenatal care provided by public service	--	1.22 (1.06-1.40)	1.21 (1.05-1.41)	1.34 (1.17-1.55)
Vaginal delivery	--	--	1.11 (0.96-1.28)	--
Birth in a Baby-Friendly Hospital	--	--	0.82 (0.72-0.96)	0.85 (0.73-0.99)
Guidance on breastfeeding received at hospital	--	--	0.80 (0.69-0.93)	0.80 (0.68-0.92)
Breastfeeding within first hour of life	--	--	0.99 (0.87-1.14)	--
Male baby	--	--	--	1.01 (0.90-1.14)
Mother partner’s appreciation for breastfeeding	--	--	--	0.62 (0.48-0.79)
Mother working outside the home	--	--	--	1.73 (1.53-1.95)
Mother limiting the number of nighttime breastfeeds	--	--	--	1.58 (1.11-2.23)
Presence of cracked nipple	--	--	--	2.54 (2.06-3.13)
Use of a pacifier	--	--	--	1.40 (1.14-1.71)

*HR (95% CI): hazard ratio (95% confidence interval).

## Discussion

### Risk factors for early EBF cessation

Of the 19 variables tested in this study, 9 showed an association with early discontinuation of EBF, i.e., before the child completed 6 months of life. Two of these variables – limiting the number of nighttime feeds at the breast and mother partner’s appreciation for breastfeeding – had not been previously evaluated in Brazilian studies, according to information from a systematic review including only studies with representative samples and with adjustment for possible confounding factors [20].

Mothers who reported limiting nighttime breastfeeds presented a 58% greater risk of early EBF cessation. This finding corroborates the association found in a survey conducted in Dubai between a lower number of nighttime breastfeeds and discontinuation of exclusive and predominant breastfeeding [24]. EBF on demand, day and night, as currently recommended, may cause physical fatigue in women, particularly those who have little or no support, and may contribute to limiting the number of breastfeeds during the night, which is also the time when prolactin levels are physiologically higher. This situation may result in a lower milk supply, and consequently, breastfeeding supplementation with other foods [25]. In addition, limiting the number of nighttime feeds at the breast may be an indicator of difficulties with breastfeeding or even a desire of the mother to wean the child. We have no knowledge of studies other than the one conducted in Dubai that have analyzed this variable as a determinant of EBF.

Another variable that had not been previously included in Brazilian studies on determinants of EBF was mother partner’s appreciation for breastfeeding. This factor behaved as a protector against discontinuation of EBF during the first 6 months of life. Women reporting that their partners showed appreciation for breastfeeding had a 38% lower risk of presenting the outcome. This finding reinforces the role of the father or partner as a facilitator in starting and maintaining breastfeeding, by encouraging and supporting the mother [12].

Three other variables that had only been investigated once as possible determinants of EBF in Brazil [20] showed associations with the outcome in the present study, namely, guidance on breastfeeding received at the maternity hospital; presence of cracked nipples; and type of service providing prenatal care.

In the present study, women who received guidance on breastfeeding at the maternity hospital presented a 20% lower risk of abandoning EBF during the first 6 months. This behavior had already been reported for the same population; in that previous study, guidance on breastfeeding received in the hospital was associated with a 34% lower risk of discontinuation of EBF during the first month after delivery [26]. The WHO recommends postpartum breastfeeding counseling, ranging from practical help and guidance on breastfeeding techniques to psychological support and guidance about myths and taboos [18]. An Australian study has shown that not attending childbirth education was negatively associated with feeding any breast milk (exclusively or partially) at 6 months [19]. Other studies also found that breastfeeding education is significantly associated with EBF practice [4,15,16]. Combined individual and group counseling appeared to had a greater impact than individual or group counseling alone [16].

The present investigation also showed that cracked nipples were associated with a 2.4-fold greater risk of discontinuing EBF within the first 6 months of life. The only Brazilian study previously investigating this variable did not find any association between nipple trauma and duration of EBF [27]. Methodological differences between that study and ours may explain the discordant results. Whereas Santo et al. considered as nipple trauma only those lesions observed during physical breast examination in the maternity hospital (blisters, ecchymosis, marks, and cracks), in our cohort this variable was self-reported and covered the whole follow-up period. Studies conducted in other countries have emphasized that nipple trauma may increase the risk of early discontinuation of breastfeeding [14,24]. This is a very important finding, given the magnitude of the association between cracked nipples and early discontinuation of EBF and the high incidence of nipple trauma. In a study conducted in southern Brazil, almost half of the women presented nipple trauma during the maternity stay, a condition attributed to the high prevalence of poor breastfeeding techniques [28]. Interventions to improve breastfeeding techniques both in the hospital and at primary care settings should be considered without delay.

The situation of the variable type of prenatal care provided (private or public service) was similar to that of presence of cracked nipples. No association with duration of EBF was reported in the single previous study that evaluated this variable [29], even though an association was found in our cohort. Again, it is important to acknowledge methodological differences between the studies: while we evaluated the type of service where prenatal care was provided (public vs. private), França et al. focused on whether the mother had access to private facilities, not giving information about the type of service providing prenatal care [29].

Our pregnant women whose prenatal care was provided by public services had a 34% higher risk of abandoning EBF within the first 6 months. This finding challenges healthcare policy makers and professionals to reassess the type of prenatal care that is being delivered to women at the public healthcare system of the municipality studied. Pregnancy and delivery are unique opportunities for providing assistance to women in making decisions regarding how to feed their children – opportunities that cannot be wasted. If, on the one hand, pregnant women who use public healthcare services are at a disadvantage regarding the duration of EBF, on the other they can benefit from using hospitals accredited with the Baby-Friendly Hospital Initiative – the accreditation of public hospitals in Brazil has been on the rise. In the present study, children who were born in a Baby-Friendly Hospital had a 15% lower risk of early discontinuation of EBF. The Baby-Friendly Hospital Initiative comprises a set of standards and routines [30] that have been correlated with better breastfeeding rates; the initiative has been considered a determinant of the positive evolution of breastfeeding rates both in Brazil [31-33] and worldwide [3,30].

The three other variables associated with early discontinuation of EBF in our study (low maternal educational level, mother working outside the home, and use of a pacifier) had been widely explored in both Brazilian and international studies. Maternal schooling level has been the factor most widely studied in Brazilian investigations (it was present in 18 of the 21 studies included in the systematic review) [20]. Eleven studies showed an association between maternal schooling level and duration of EBF, and the majority found that women with lower schooling levels were at a higher risk of discontinuing EBF [20]. Our finding that women with up to 8 years of schooling had a 34% higher risk of presenting the outcome corroborates those previous reports. A nationwide study conducted by the Brazilian Ministry of Health confirmed this association, observing that mothers with higher schooling levels exclusively breastfed for longer times [11]. In the international setting, the association between maternal educational level and EBF also varies depending on the locality assessed. A low educational level sometimes acts as a risk factor for EBF cessation [34,35] and sometimes as a protective factor [5,7,34,35].

Mother working outside the home was the second variable most commonly evaluated in Brazil (in 17 studies) [20], but only three studies identified it as a determinant of shorter duration of EBF [36-38]. The greater likelihood of discontinuation of EBF observed among women who worked outside the home in our sample may have had some relationship with the work patterns observed in the city studied. Because this is a region with a high poverty rate, it is possible that more women were in informal employment in our sample (maids, cleaners, and street vendors, among other activities) and therefore did not have the right to maternity leave. In Brazil, mothers with a formal work contract have the right to 120 days of maternity leave; this period has been extended to 180 days for public employees and in some private companies [39]. In this regard, it is important to bear in mind that the median duration of EBF in Brazil is only 1.4 month, i.e., much shorter than the duration of the maternity leave; this may explain why working outside the home was not a determinant of EBF in most studies. In our study, however, median duration of EBF was almost 3 months, a time at which many women without the right to maternity leave would already have returned to work. Similarly, other studies in different countries such as Honduras [34], Canada [5], Timor-Leste [6] and Ethiopia [4], have shown that women returning to work were less successful in maintaining EBF than their counterparts who did not return to work. In addition, in Mexico, most women interrupted EBF long before returning to work, a fact that may explain the absence of this association in that country [34].

Lastly, the association between pacifier use and duration of EBF also deserves to be discussed. This is the association most frequently found in the literature, and was present in 12 of the 13 Brazilian studies in which this factor was evaluated [20]. Our study also confirmed it. The international scenario does not differ from the Brazilian one with regard to the negative influence of the use of a pacifier on duration of EBF [17,19]. For instance, a previous Australian cohort study has found an association between introducing a pacifier before the 10th week of life and early EBF interruption [19]. Even though this association is unequivocal, a definitive explanation for it is still lacking. Using a dummy/pacifier may reduce the frequency of breastfeeds, alter the baby’s oral dynamics [40], or cause nipple confusion; [41] it may also be a sign of difficulty with breastfeeding or the mother’s desire to wean her child [42].

### The situation of EBF in Feira de Santana

According to the results of this study, the situation of EBF in Feira de Santana is better than the overall situation in Brazil. Whereas, in Brazil, the estimated median duration of EBF is 1.4 month, in Feira de Santana it was almost 3 months; [9] also, the prevalence of EBF at 30 and 180 days in Brazil is 60.7% and 9.3%, respectively, compared to 89.6% and 11.3% in our cohort from Feira de Santana [11].

### Methodological considerations

One of the strengths of this study is its design: this was a prospective cohort with a probabilistic sample, with subjects who are representative of the city and low rate of loss to follow-up. Another merit of the study is the investigation of variables that had received little or no attention in previous literature.

The theoretical hierarchical model adopted made it possible to evaluate how variables in the same group compete with each other and how more proximal variables may mediate the effects of variables in a preceding group [43]. For example, we observed that the magnitude of the associations substantially changed after adjustment for proximal models. Also, it was possible to show that variables in the proximal level were overall the ones most strongly associated with the outcome, and that, at a collective level, association strength increased as the proximity of variables to the outcome increased.

Among the methodological limitations of the present study, the high volume of self-reported data, which were not directly measured (e.g., mother partner’s appreciation for breastfeeding and guidance on breastfeeding received at the maternity hospital, among other variables), should be emphasized. As is the case in any investigation of determinants, the model here used was only partially capable of explaining the outcome.

## Conclusions

The present study confirmed that the determinants of EBF duration are multiple, variable, and dependent on the population studied. We believe that our findings add to the existing literature by expanding our knowledge of determinants of EBF, including factors that had received little or no attention before. Furthermore, the results here described may provide a basis for the development of public policies that promote, protect and support EBF in the municipality investigated.

## Abbreviations

EBF: Exclusive breastfeeding; SPSS: Statistical package for the social sciences; WHO: World health organization.

## Competing interests

The authors declare that they have no competing interests.

## Authors’ contributions

TOV conceived the study hypothesis, performed the analysis and drafted the paper. GOV conceived the study hypothesis, designed and conducted the cohort study. NFO carried out the analysis and interpretation of data. CMCM provided statistical guidance and contributed to manuscript writing. ERJG provided statistical guidance and substantial contributions to data interpretation and writing. LRS assisted with study design, provided substantial contributions to data interpretation, and supervised manuscript writing. All authors helped to interpret the findings, reviewed and approved the final draft.

## Pre-publication history

The pre-publication history for this paper can be accessed here:

http://www.biomedcentral.com/1471-2393/14/175/prepub
